# Large-scale microtubule networks contract quite well

**DOI:** 10.7554/eLife.14076

**Published:** 2016-02-12

**Authors:** Julio M Belmonte, François Nédélec

**Affiliations:** Cell Biology and Biophysics Unit, European Molecular Biology Laboratory, Heidelberg, Germany; Cell Biology and Biophysics Unit, European Molecular Biology Laboratory, Heidelberg, Germanynedelec@embl.de

**Keywords:** active matter, cytoskeleton, microtubules, dynein, *Xenopus*

## Abstract

The quantitative investigation of how networks of microtubules contract can boost our understanding of actin biology.

**Related research article** Foster P, Fürthauer S, Shelley M, Needleman D. 2015. Active contraction of microtubule networks. *eLife ***4**:e10837. doi: 10.7554/eLife.10837

**Image** Stabilized microtubules organize into networks of star-shaped structures called asters

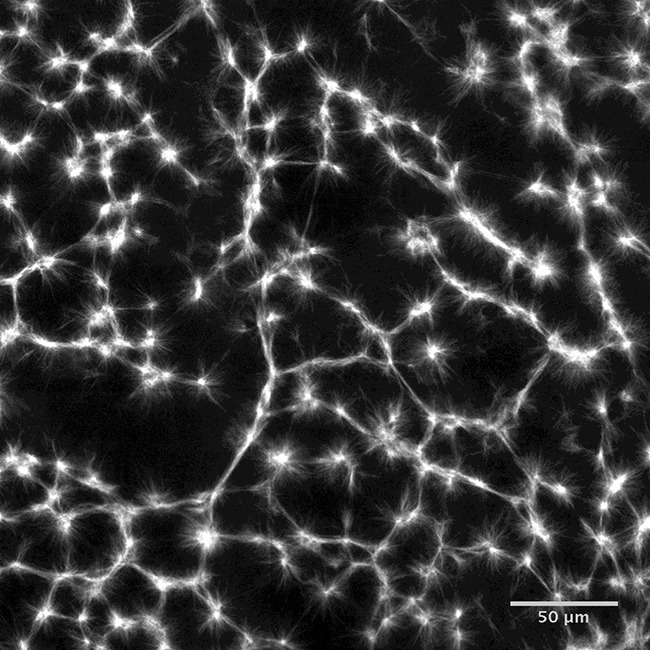

The cytoskeleton of a cell plays many important roles, such as giving the cell its shape and organizing its contents. The filaments that make up the cytoskeleton assemble from protein monomers found in the cell’s cytoplasm. Two particularly important filament types for eukaryotic cells are actin filaments and microtubules, which both have crucial roles during various stages of cell division. For example, the mitotic spindle, which is essential for chromosome segregation, is made of microtubules. Motor proteins (for example myosin, kinesin and dynein) often work with these filaments to transport material across the cell and to form contracting structures such as muscles.

In the past decades, much effort has gone into characterizing the properties of microtubules, actin filaments and motor proteins, and their most important properties have probably been discovered already. However, we need a much better understanding of how all these components work together. Now, in eLife, Peter Foster, Sebastian Fürthauer, Michael Shelley and Daniel Needleman report the first quantitative study of an important process in this field of research – the contraction of microtubule networks (Foster et al., 2015).

Instead of relying on purified proteins to study how microtubules and motors organize (see, for example, Hentrich and Surrey, 2010), Foster et al. used extracts from frog eggs. These provide a more natural mixture of components and are commonly used to study the assembly of spindles (Sawin and Mitchison, 1991). They also performed the experiments in millimeter-wide channels, allowing them to finely control the overall geometry of the network. In all the experiments, drugs were used to promote the formation of stable microtubules and to prevent actin monomers assembling into filaments.

The microtubules initially formed in random configurations, and under the action of motor proteins assembled into star-shaped structures called asters, as previously reported (Hentrich and Surrey, 2010). The whole microtubule network then slowly contracted.

To clarify how these processes occurred, Foster and colleagues – who are based at Harvard University and New York University – used drugs to separately inhibit the activity of kinesin and dynein. This demonstrated that dynein accounts for 96% of the active stress in microtubule networks. Remarkably, carefully analyzing the contraction of the microtubule network also provided insights into actin biology. How is this possible?

While microtubule and the actin cytoskeleton are similar in many ways, there are important differences in the structures they form and the behaviors they display in vivo. Microtubules tend to form structures such as radial arrays because the filaments are few and tend to be straight due to their high rigidity. Moreover, since microtubules are often as long as the cell, the cell simply does not provide enough space to build the large microtubule networks that would be necessary for observing contraction. On the other hand, contraction is a common feature of actin networks, which can be made of many relatively short filaments that are 200 times more flexible than microtubules. These considerations reflect the fact that the behavior of a network is often largely a matter of scale: indeed, networks of filaments are usually analyzed in terms of filament length, the density of the filaments, and the overall size of the network (Lenz et al., 2012).

In the past, researchers have studied the contraction of actin networks at the micrometer scale. Now, Foster et al. were able to monitor the contraction of microtubule networks in millimeter-wide channels. Looking at the contractile behavior of filament networks in different regimes is especially valuable, because different contraction mechanisms are thought to operate at different scales. Actin network contractility is thought to require the bending of filaments, whereas microtubule contractility would rely on molecular motors holding tight to the ends of the microtubules (Figure 1). The ability to compare these two systems should improve our understanding of the general principles of contractility, and thus contribute to actin biology.

**Figure 1.**
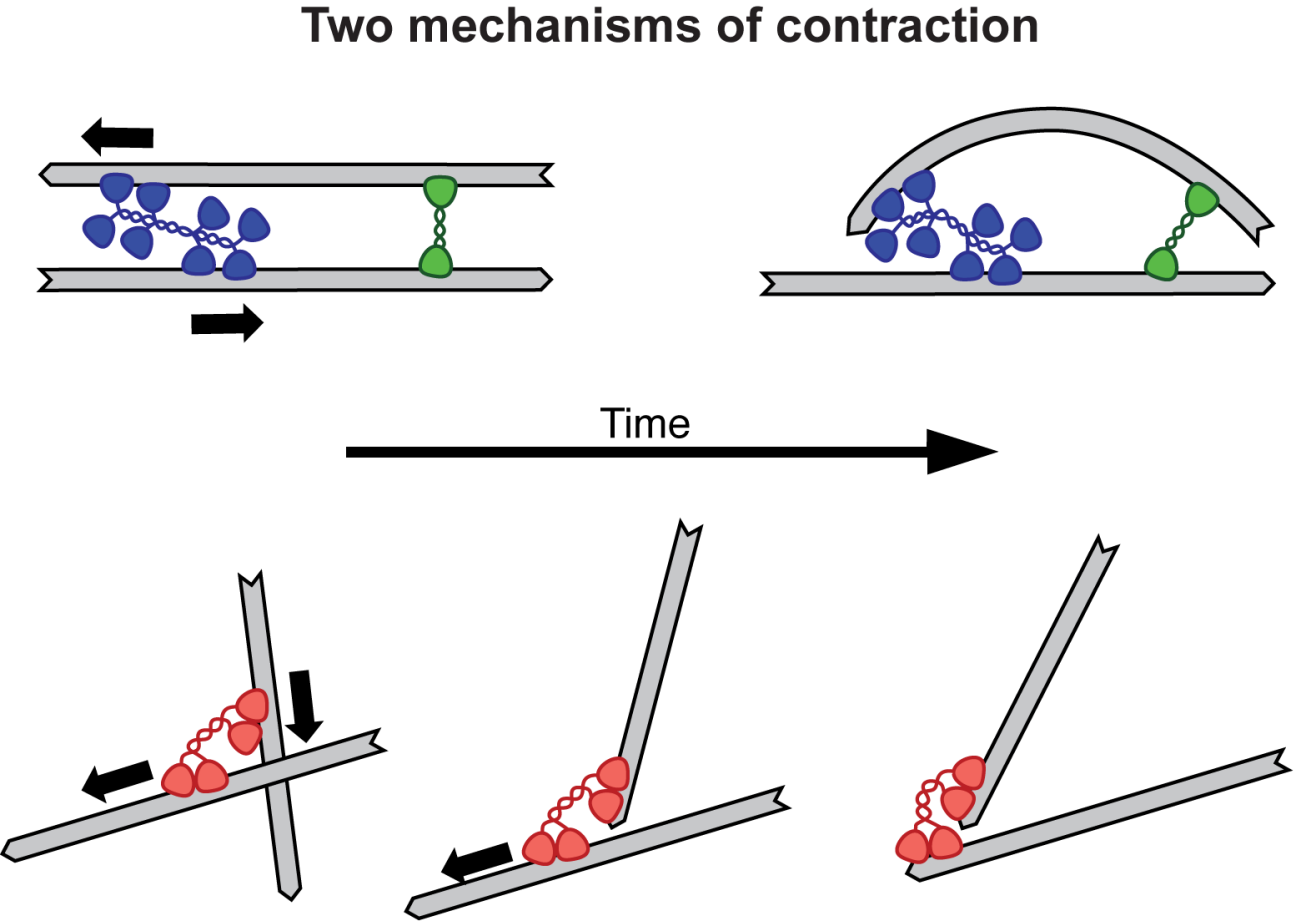
Two mechanisms for contraction: buckling and end clustering. Top: When two anti-parallel actin filaments are bridged by a myosin motor (blue) and a crosslink (green), their relative movement forces one filament to buckle, resulting in the contraction of the network. Bottom: Microtubule contraction seems to depend on the affinity of dynein motors (red) for the ends of the filaments. For a recent review on the topic of contraction, see Clark et al., 2014.

Foster et al.’s approach may also teach us more about how mitotic spindles form. The molecular motor dynein, which induces the bulk contraction of large random networks, is also thought to help form the focused poles of the spindle. Specifically, contractions driven by dynein motors likely help the spindle to adopt the correct shape. Thus by carefully quantifying this contraction process, Foster et al. have likely given us some of the parameters needed to create accurate models of the mitotic spindle. For instance, the extract always contracted to the same final density, which is surprisingly similar to the density of the mitotic spindle. Future research could investigate the mechanism responsible for this density limit.

A remarkable aspect of the study is that Foster et al. could fit the bulk properties of the contraction with a simple active gel theory, using just four parameters. For example, the theory can explain how the microtubule density varies at the edge of the network and how the rate of contraction depends on the overall size of the network. This advance in our knowledge of cytoskeletal network contractility was only possible through a tight interplay between experiments and theory.
